# Supplementary figures and images for: Genome-scale requirements for dynein-based transport revealed by a high-content arrayed CRISPR screen
Source: J Cell Biol. 2024 Mar 6;223(5):e202306048. doi: 10.1083/jcb.202306048 (PMC10916854; doi:10.1083/jcb.202306048)

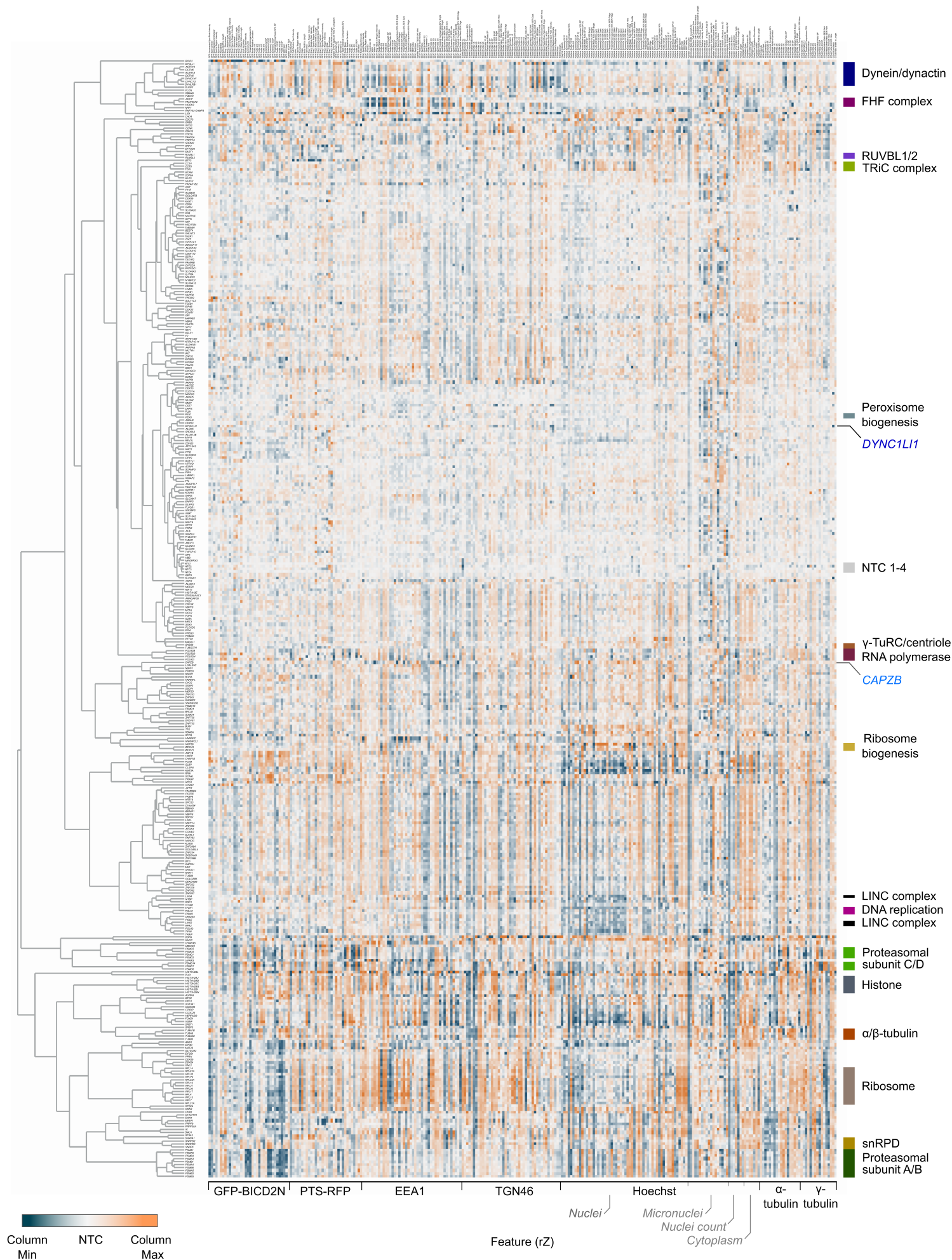

Supplement: Data S1 — shows a high-resolution, explorable version of the phenotypic heatmap. [file JCB_202306048_DataS1.pdf]
