# Supplementary material for: Genome-scale requirements for dynein-based transport revealed by a high-content arrayed CRISPR screen
Source: J Cell Biol. 2024 Mar 6;223(5):e202306048. doi: 10.1083/jcb.202306048 (PMC10916854; doi:10.1083/jcb.202306048)
Supplement: Data S2 — shows image acquisition and analysis workflow for genome-wide CRISPR screen. [file JCB_202306048_DataS2.pdf]

IMAGE ACQUISITION AND ANALYSIS WORKFLOW FOR GENOME-WIDE CRISPR SCREEN

Image acquisition

Image acquisition was performed on the Cell Voyager 8000 (Yokogawa) with a 20X water objective (20x W-Y1; 1.0 NA) with 2x2 binning and 4 fields of view across each well. The fluorescence channels were as follows:

Stain or fluorescent molecule	Labels	Laser	Laser power	Exposure time	Acquisition
Hoechst	Nucleus	405 nm	100%	40 ms	BP445/45
GFP-BICD2N-FRB	Dynein adaptor	488 nm	70%	250 ms	BP525/50
PTS-RFP-FKBP	Peroxisomes	561 nm	20%	100 ms	BP600/37
α-Tubulin	Microtubules	488 nm	70%	200 ms	BP646/132
EEA1	Early endosomes	640 nm	100%	100 ms	BP676/29

Workflow description

Individual building blocks were generated through Columbus 2.9.1 (Perkin Elmer). The sections below describe the aim (e.g. find nucleus) and details of each building block. The following details are included:

1. Input

Channel: Marker of interest (e.g. Hoechst)

Population: Cellular populations of interest based on a pre-defined gate (e.g. 'initial population' – all the detected nuclei within one frame of view, see building block 1)

Region: Target cellular compartment selected for analysis (e.g. nucleus)

2. Method

Based on the selected channel, population and region, Columbus methods were applied to the population of interest (e.g. all detected nuclei) or used to measure the region of interest (e.g. nucleus area). A brief description of the method is provided based on the Columbus analysis handbook (2.9.1). Gating (e.g. based on intensity thresholds) was optimised based on multiple iterations of manual tuning of parameters using randomly selected images of non-targeting control (NTC) cells coupled to visual evaluation of detection or segmentation of desired objects.

Whenever appropriate, further evaluation was performed by comparing the results with NTCs to positive controls for endpoints, including *crPLK1* (e.g. for proportion of viable cells). For example, when developing parameters for detecting peroxisomes or early endosomes, values

were iteratively modified based on their performance with a set of images treated with NTC crRNAs, *crLIS1* or nocodazole.

3. Output

Upon application of the Columbus method (e.g. 'Standard for 2D analysis') to the input, outputs such as regions, populations or quantitative measurement were used for further analysis.

4. Example of segmented images

An example image is included whenever segmentation is applied. The intensity and contrast of these images have been manually adjusted for display purposes. Unless specified, the example of segmented images shown was a non-targeting control that was selected at random (Plate ID: 1090959889; Well: B23; Field: 2). Where applicable, an image of *crLIS1* is shown for comparison (Plate ID: 1090959889; Well: B21; Field: 2).

Building blocks

1. Find Nuclei

Aim: Detect regions in the image corresponding to individual nuclei

Input	Method	Output	Example of segmented images
Channel: Hoechst<br><br>Region: None	Method: B<br><br>Common Threshold: 0.05<br><br>Area:>33 µm<br><br>Splitting Coefficient: 7<br><br>Individual Threshold: 0.15<br><br>Contrast: >0.1	Region: Nucleus<br><br>Population: Initial population<br><br>Feature(s): Number of cells initial	NTC:<br>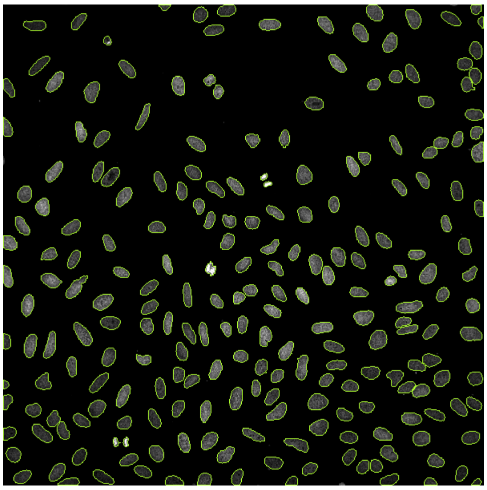<br>Green border: segmented nucleus

Method B was selected for its general robustness for Hoechst-stained images.

Common threshold: The lower level of pixel intensity that is classed as belonging to nuclei

Area: Lower limit of area of a detected nucleus

Splitting coefficient: Determining whether a large object is split into two or more smaller objects

Individual threshold: Determining the intensity threshold for each object. A brighter object has a higher absolute intensity threshold determining its border than a dimmer object.

Contrast: Lower contrast value threshold for classification of nuclei

2. Find cytoplasm

Aim: Detect regions around individual nuclei belonging to the cytoplasm

Input	Method	Output	Example of segmented images
Channel:<br>α-Tubulin<br><br>Nuclei:<br>Initial population (from building block 1)	Method: A<br>Individual threshold: 0.05	Region:<br>Cytoplasm	NTC:<br>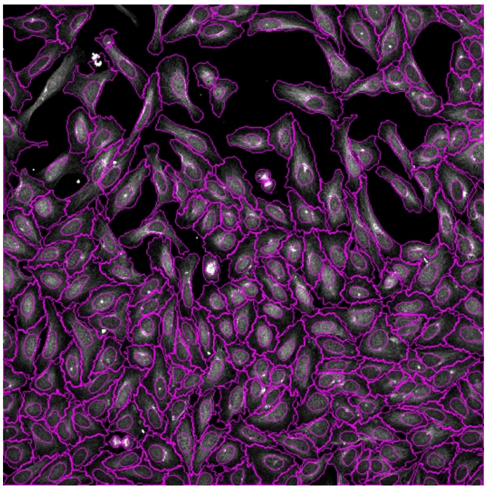<br>Magenta: segmented cytoplasm

Method A was selected as the most robust method for defining the cytoplasm.

3. Remove border objects

Aim: Exclude cells from further analysis that have nuclei with an associated cell region that overlaps with the image border

Input	Method	Output	Example of segmented images
Nuclei:<br>Initial population (from building block 1)	Method:<br>Common Filters<br><br>Remove border objects region:<br>Cell	Population:<br>Initial population removed border<br><br>Feature(s):<br>Number of cells removed border	NTC:<br>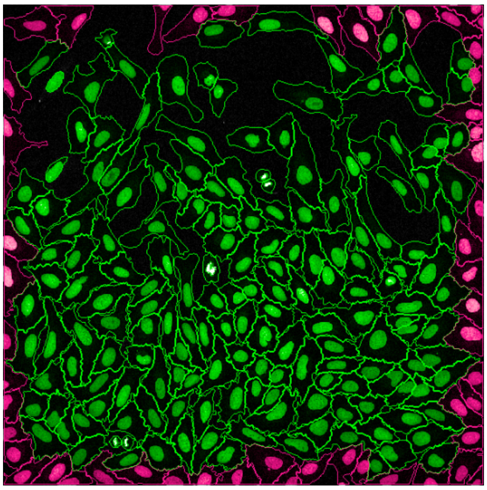<br>Green – selected;<br>Magenta - excluded

4. Calculate nucleus morphology properties

Aim: Quantify nucleus morphology to gate for viable cells

Input	Method	Output	Example segmented images
Nuclei: Initial population removed border (from building block 3)<br><br>Region: Nucleus	Method: Standard	Feature(s):<br>• Area<br>• Roundness<br>• Width<br>• Length<br>• Ratio Width to Length	N/A

5. Calculate cytoplasm morphology properties

Aim: Quantify cytoplasm morphology to gate for viable cells

Input	Method	Output	Example segmented images
Nuclei: Initial population removed border (from building block 3)<br><br>Region: Cytoplasm	Method: Standard	Feature(s):<br>• Area<br>• Roundness<br>• Width<br>• Length<br>• Ratio Width to Length	N/A

6. Select cell population

Aim: Select for viable cells by excluding debris and mitotic cells

Input	Method	Output	Example segmented images
Nuclei: Initial population removed border (from building block 3)	Method: Filter by property<br><br>Nucleus area (μm^2): ≥ 208<br>Roundness: ≥ 0.86<br>Cytoplasm area (μm^2): ≥ 400	Population: Viable cells<br><br>Feature(s):<br>• Number of viable cells<br>• Proportion of viable cells	NTC:<br>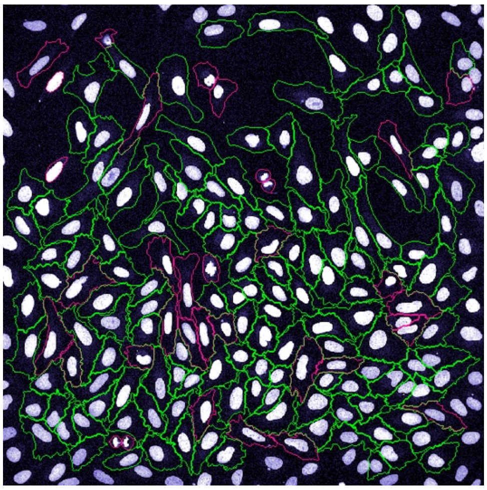<br><br>*crPLK1*:<br>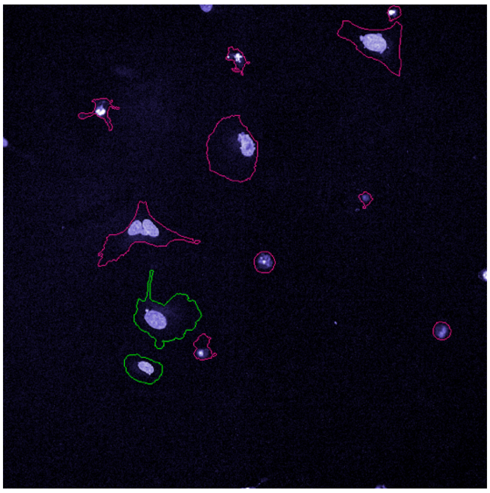<br>Green – selected;<br>Magenta - excluded

7. Resize cytoplasm region for perinuclear spots

Aim: As peroxisomes spots are often located close to the nucleus due to their clustering at the MTOC, the segmented cytoplasm is not sufficient to accurately account for these spots. The cytoplasm is therefore ‘resized’ inward to account for these spots and enable detection.

Input	Method	Output	Example segmented images
Nuclei: Viable cells (from building block 6)<br><br>Region: Cytoplasm	Method: Resize region [%]<br><br>Outer border: -20 %<br>Inner border: 75%	Region: Cytoplasm resize	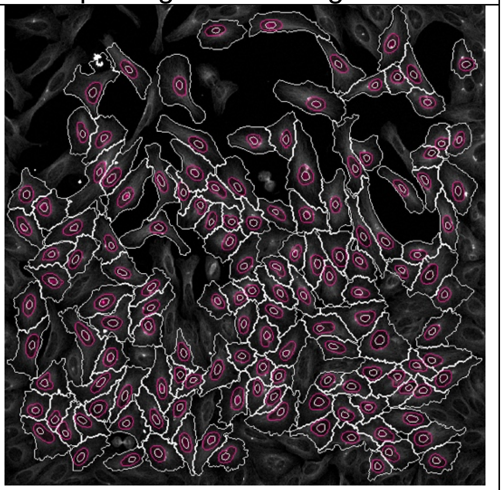<br>Magenta: Nucleus boundary<br>White: The ‘resized’ cytoplasm expands inward towards the center of the nucleus

The border of the cytoplasm region is tuned by the ‘Outer border’ and ‘Inner border’.

When the value of the ‘Inner border’ is infinity, then the ‘Outer border’ alone determines the border of the newly created region.

Increasing a tuning parameter will cause an inward shift of the border that is adjusted.

0% - corresponds to the original border.

100% - corresponds to the inner centre of the original region.

8. Calculate GFP-BICD2N-FRB intensity

Aim: Calculate average GFP-BICD2N-FRB pixel intensity within the resized cytoplasm (from building block 7)

Input	Method	Output	Example segmented images
Channel: GFP-BICD2N-FRB<br><br>Nuclei: Viable cells (from building block 6)	Method: Standard	Output property: Mean<br><br>Output features: GFP-BICD2N FRB-intensity initial population	N/A

Region:<br>Cytoplasm<br>resize			

9. Calculate PTS-RFP-FKBP intensity

Aim: Calculate average GFP-BICD2N-FRB pixel intensity within the resized cytoplasm

Input	Method	Output	Example segmented images
Channel:<br>PTS-RFP-<br>FKBP<br><br>Nuclei:<br>Viable<br>cells<br>(from<br>building<br>block 6)<br><br>Region:<br>Cytoplasm<br>resize	Method: Standard	Output property:<br>Mean<br><br>Output features:<br>PTS-RFP-FKBP<br>intensity initial<br>population	N/A

10. Select cell populations that co-express GFP-BICD2N-FRB and PTS-RFP-FKBP

Aim: Select population with expression of both GFP-BICD2N-FRB and PTS-RFP-FKBP for further quantification

Input	Method	Output	Example segmented images
Nuclei:<br>Viable<br>cells<br>(from<br>building<br>block 6)	Method: Filter by<br>property<br><br>(F1) Mean Intensity<br>GFP: ≥ 32<br><br>(F2) Mean intensity<br>RFP: ≥ 9<br><br>Boolean<br>Operations: F1 and<br>F2 (to gate for cell<br>population that<br>fulfils criteria of<br>both F1 (GFP<br>intensity) AND F2<br>(RFP intensity))	Population:<br>GFP and RFP<br>positive cells	Green bordered cell = selected<br>Magenta bordered cell = excluded<br><br>Image from both channels:<br>Green spots = GFP-BICD2N-FRB<br>Magenta spots = PTS-RFP-FKBP<br>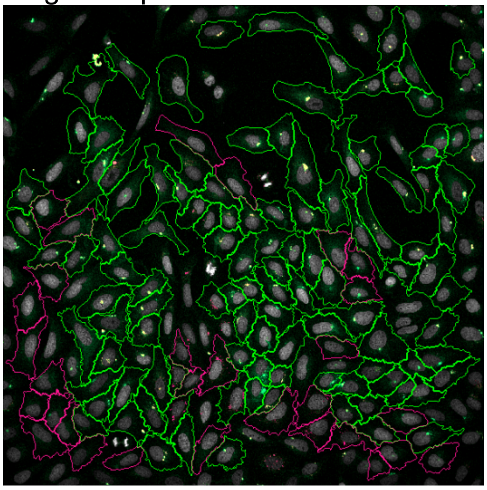

			Images from individual channels:<br>GFP-BICD2N-FRB<br>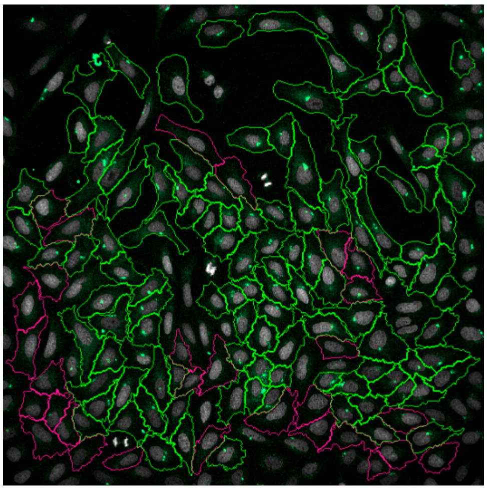<br>PTS-RFP-FKBP<br>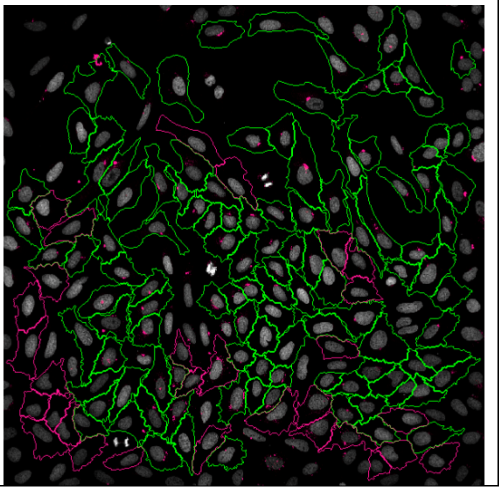

11. Find total GFP spots

Input	Method	Output	Example segmented images
Channel: GFP-BICD2N-FRB<br><br>Nuclei: GFP and RFP positive cells (from building block 10)<br><br>Region: Cytoplasm resize	Method: B<br><br>Detection sensitivity: 0.6 (determines how intense a spot must be to be detected: at lowest value (0.0) only extremely bright spots are detected, whereas at highest value (-1.0) extremely weak spots are also detected	Population: GFP spots<br><br>Output features:<br>• Number of GFP-BICD2N-FRB spots<br>• GFP-BICD2N-FRB relative spot intensity	GFP-BICD2N-FRB channel:<br>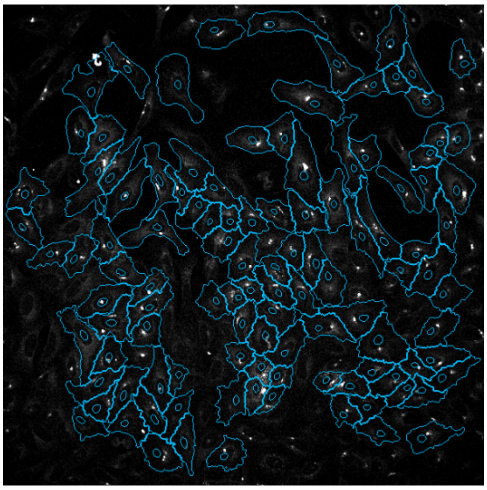<br>Blue bordered cell = GFP and RFP positive cells

	Spitting sensitivity: 0.835 (Determines split-or-merge of adjacent spots: at the lowest value (0.0), detected regions if connected are considered a single spot, whereas at highest value (1.0) there is a maximum number of split lines)		Detected GFP-BICD2N-FRB spots: 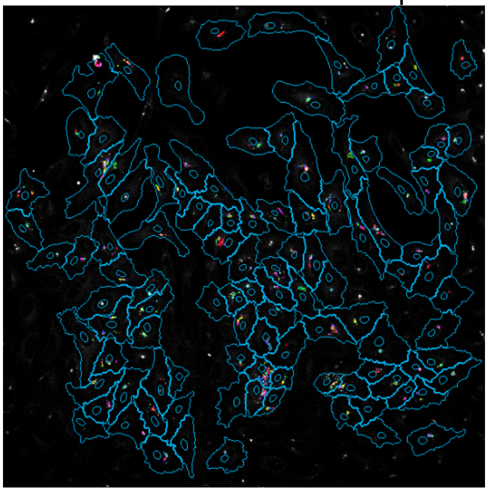 Rainbow-coloured: individual spot

Method B: Estimates the background of the image and adapts to spot size (i.e. does not require a specific radius size to detect a spot).

Relative spot intensity: The ratio of the 'corrected spot intensity' and the 'mean spot intensity'.

Corrected spot intensity: 'Mean spot intensity' minus 'spot background intensity'

Spot background intensity: Mean intensity of the spot border

Because GFP spots are not readily detected until they are clustered, increased GFP spot number is a proxy for peroxisome clustering.

12. Find total RFP spots

Input	Method	Output	Example segmented images
Channel: PTS-RFP-FKBP<br><br>Nuclei: GFP and RFP positive cells (from building block 10)<br><br>Region: Cytoplasm resize	Method: D<br><br>Detection sensitivity: 0.75<br><br>Spitting sensitivity: 0.04<br><br>Background correction: 0.5 (for estimation and subtraction of background (0.5 = default): at the lowest value (0.0) only very gradual changes in the background intensity are allowed (may result in undercorrection	Population: RFP spots	PTS-RFP-FKBP channel: 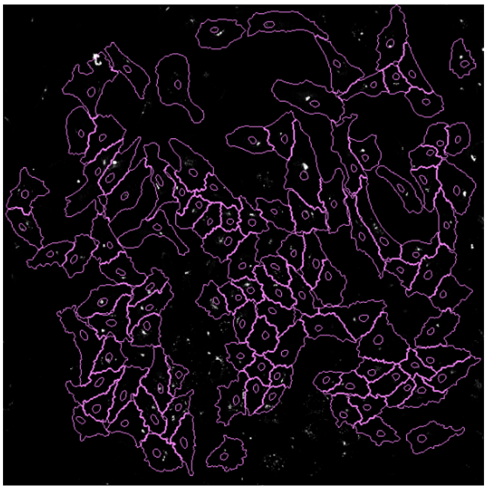 Magenta bordered cell = GFP and RFP positive cells

	that picks up background signal as spots), whereas at highest value (1.0), the background intensity is allowed to change very abruptly (may result in overcorrection that misses out weaker spots)		Detected PTS-RFP-FKBP spots:<br>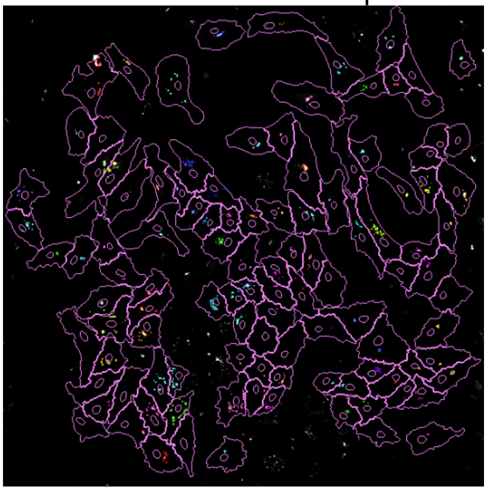<br>Rainbow-coloured: individual spot

Method D: Estimates background of image and adapts to spot size.

Method D was selected as the RFP channel has relatively higher background signal compared to GFP and therefore required a background subtraction step

Because RFP spots are readily detected when they are not clustered, decreased RFP spot number is a proxy for peroxisome clustering as multiple spots coalesce into a smaller number of spots.

13. Calculate GFP spot morphology

Input	Method	Output	Example segmented images
Channel:<br>GFP spot<br><br>Nuclei:<br>GFP and RFP positive cells (from building block 10)<br><br>Region:<br>GFP spot	Method: Standard	Features(s):<br>• Area<br>• Roundness<br>• Width<br>• Length<br>• Ratio Width to Length	N/A

14. Calculate RFP spot morphology

Input	Method	Output	Example segmented images
Channel:<br>RFP spot<br><br>Nuclei:<br>GFP and RFP	Method: D<br><br>Detection sensitivity:<br>0.75<br><br>Spitting sensitivity:<br>0.04	Features(s):<br>• Area<br>• Roundness<br>• Width<br>• Length<br>• Ratio Width to Length	N/A

positive cells (from building block 10)<br><br>Region: Cytoplasm resize	Background correction: 0.5		

15. Calculate GFP texture properties

Input	Method	Output	Example segmented images
Channel: GFP-BICD2N-FRB<br><br>Nuclei: GFP and RFP positive cells (from building block 10)<br><br>Region: Cytoplasm resize	Method: SER features<br><br>Scale: 0.65 μm<br><br>Normalisation by: Region intensity	Feature(s):<br>• SER spot<br>• SER Hole<br>• SER Edge<br>• SER Valley<br>• SER Saddle<br>• SER Bright<br>• SER Dark	Filtered images on GFP channel:<br>SER Spot:<br>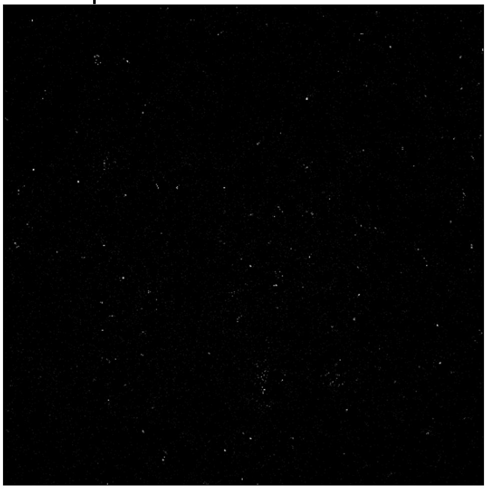<br><br>SER Hole:<br>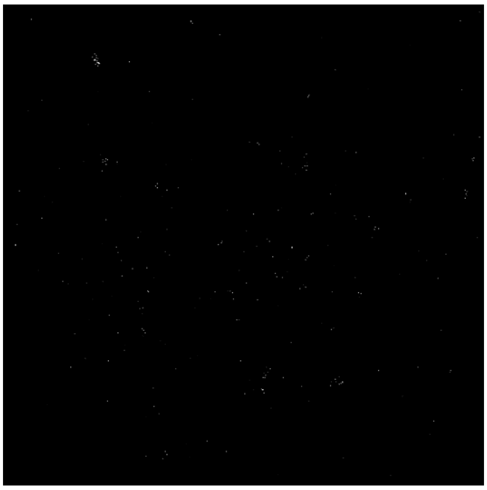

			SER Valley:<br>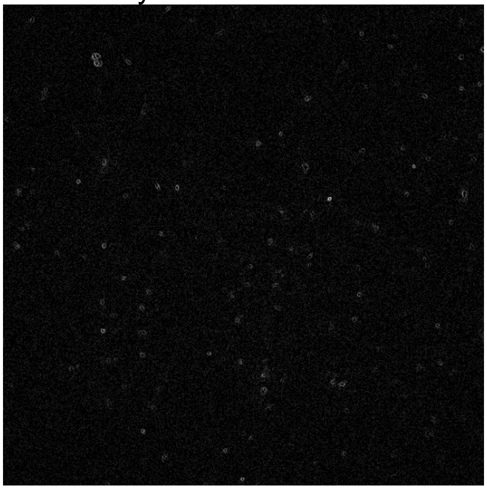<br>SER Saddle:<br>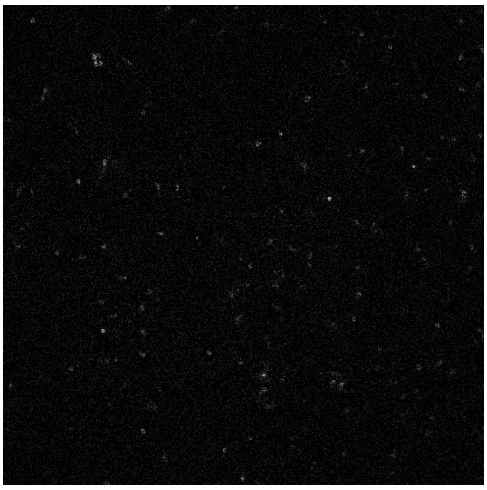<br>SER bright:<br>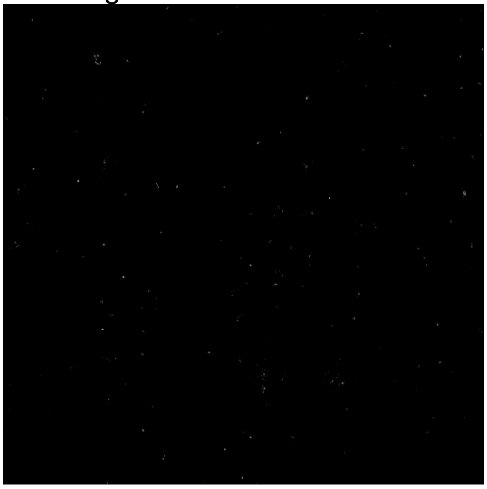

			SER dark: 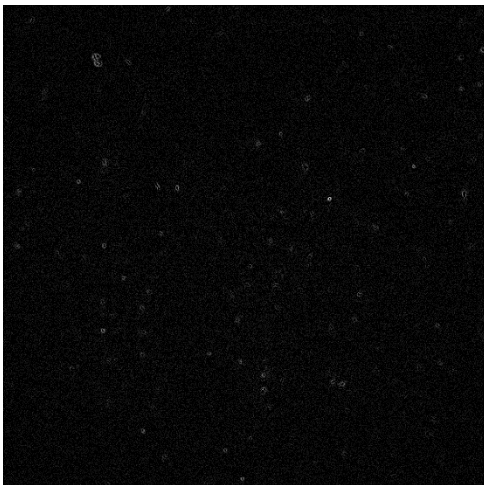

SER: acronym for spots, edges, and ridges

Scale: the characteristic length of a smoothing operation when calculating filtered images

Normalisation by region intensity: Filtered images are pixelwise divided by intensity of the original image in the corresponding object

16. Calculate RFP texture properties

Input	Method	Output	Example segmented images
Channel: PTS-RFP-FKBP<br><br>Nuclei: GFP and RFP positive cells (from building block 10)<br><br>Region: Cytoplasm resize	Method: SER features<br><br>Scale: 0.65 μm<br><br>Normalisation by: Region intensity	Feature(s):<br>• SER spot<br>• SER Hole<br>• SER Edge<br>• SER Valley<br>• SER Saddle<br>• SER Bright<br>• SER Dark	N/A

17. Resize cell region for ring regions (perinuclear ring)

Input	Method	Output	Example segmented images
Nuclei: GFP and RFP positive cells	Method: Resize region [μm]<br>Region type: Ring region<br><br>Outer border: -7 μm<br>Inner border: 7 μm	Region: Perinuclear ring	See building block 19

(from building block 10)			

A cutoff value of 7 μm was found to give less variance than other distance cutoffs when quantifying localisation ratio and perinuclear spot number in pilot analysis with cells treated with NTC, *crLIS1* or nocodazole.

18. Resize cell region for ring regions (Intermediate ring)

Input	Method	Output	Example segmented images
Nuclei: GFP and RFP positive cells (from building block 10)	Method: Resize region [μm]<br>Region type: Ring region<br><br>Outer border: -7 μm<br>Inner border: -14 μm	Region: Intermediate ring	See step 19

19. Resize cell region for ring regions (Outer ring)

Input	Method	Output	Example segmented images
Nuclei: GFP and RFP positive cells (from building block 10)	Method: Resize region [μm]<br>Region type: Ring region<br><br>Outer border: -14 μm<br>Inner border: -35 μm	Region: Outer ring	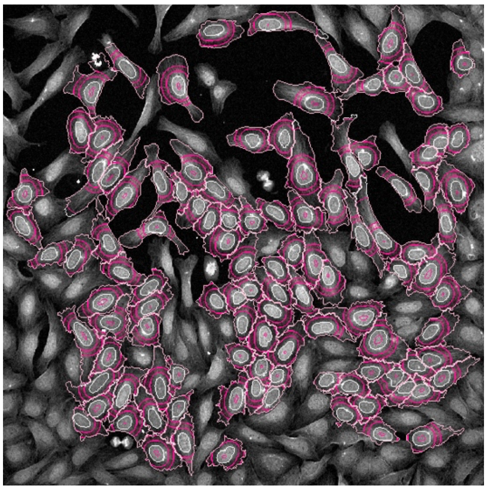<br>White border = segmented nucleus<br>Magenta border = all segmented rings – Perinuclear, Intermediate, Outer (cell periphery)

20. Find perinuclear GFP spots

Input	Method	Output	Example segmented images
Channel: GFP-BICD2N-FRB<br><br>Nuclei: GFP and RFP positive cells (from building block 10)<br><br>Region: Perinuclear ring	Method: B<br><br>Detection sensitivity: 0.6<br><br>Spitting sensitivity: 0.835	Population: Perinuclear GFP spots	NTC:<br>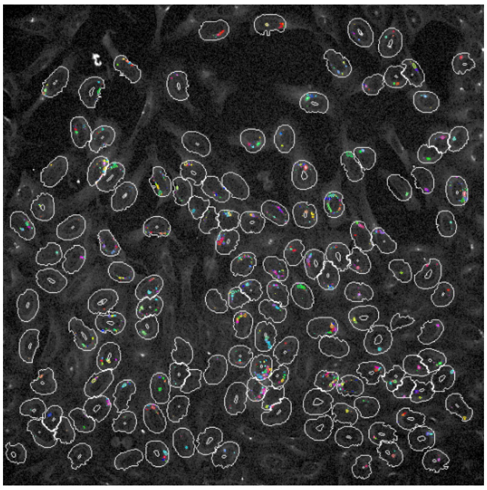<br><br>*crLIS1:*<br>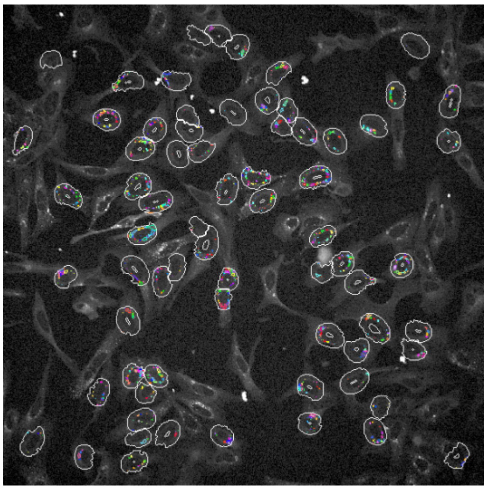<br>White border: Perinuclear ring (from building block 17)<br>Rainbow: Detected GFP spots

21. Find intermediate GFP spots

Input	Method	Output	Example segmented images
Channel: GFP-BICD2N-FRB<br><br>Nuclei: GFP and RFP positive cells	Method: B<br><br>Detection sensitivity: 0.6<br><br>Spitting sensitivity: 0.48	Population: Intermediate GFP spots	NTC:

(from building block 10)<br><br>Region: Intermediate ring			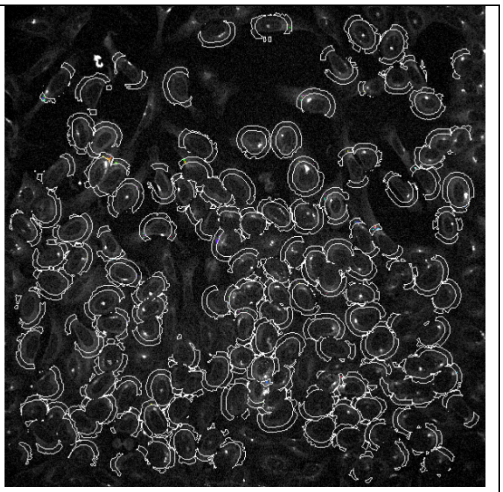<br><br>*crLIS1:*<br>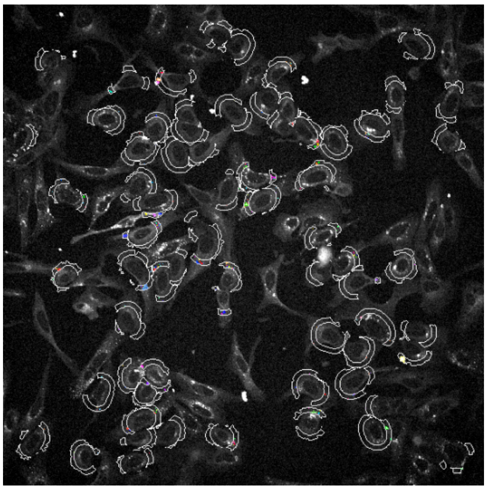<br>White border: Intermediate ring (from building block 18)<br>Rainbow: Detected GFP spots

22. Find outer GFP spots

Input	Method	Output	Example segmented images
Channel: GFP-BICD2N-FRB<br><br>Nuclei: GFP and RFP positive cells (from building block 10)<br><br>Region: Outer ring	Method: B<br><br>Detection sensitivity: 0.6<br><br>Spitting sensitivity: 0.5	Population: Outer GFP spots	NTC:<br>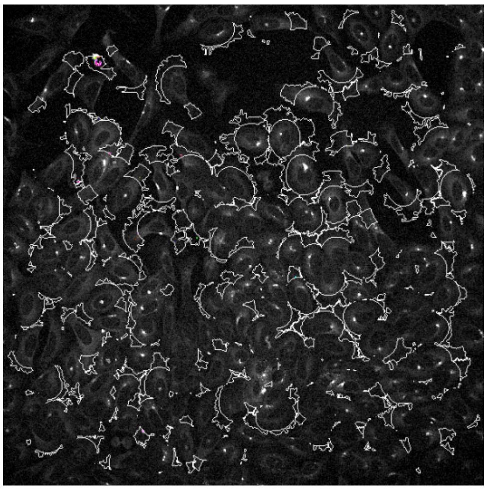

			crLIS1:<br>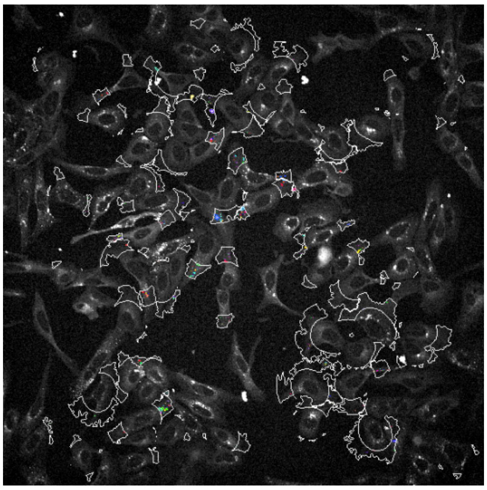<br>White border: Outer ring (from building block 19)<br>Rainbow: Detected GFP spots<br><br>[Summary of all 3 rings]<br>NTC:<br>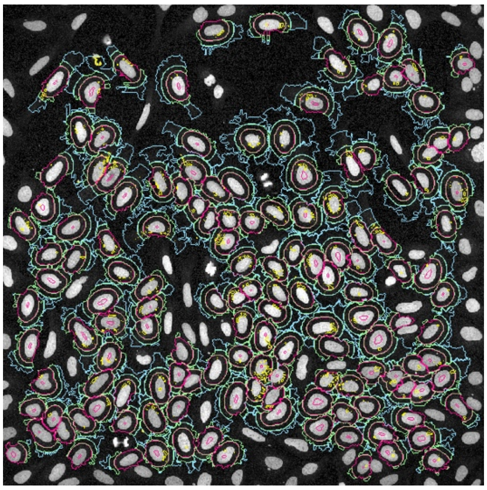<br><br>*crLIS1*:<br>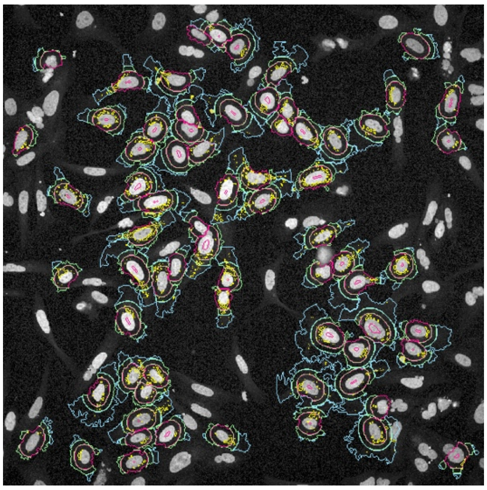<br>Coloured border: Perinuclear (Magenta), Intermediate (Green), Outer (Blue) rings

			Yellow: Detected GFP spots

23. Find perinuclear RFP spots

Input	Method	Output	Example segmented images
Channel:<br>PTS-RFP-FKBP<br><br>Nuclei:<br>GFP and RFP positive cells (from building block 10)<br><br>Region:<br>Perinuclear ring	Method: D<br><br>Detection sensitivity: 0.8<br><br>Spitting sensitivity: 0.04<br><br>Background correction: 1	Population:<br>Perinuclear RFP spots	NTC:<br>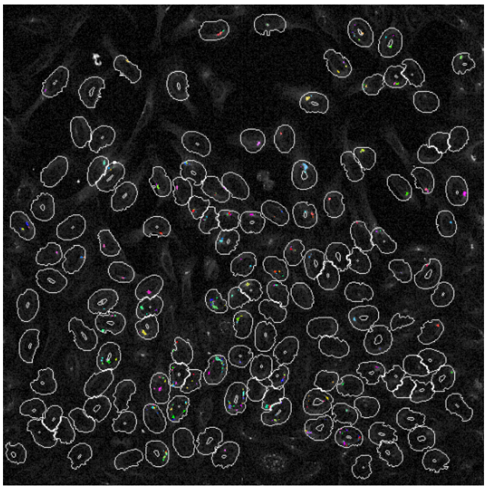<br><br>*crLIS1*:<br>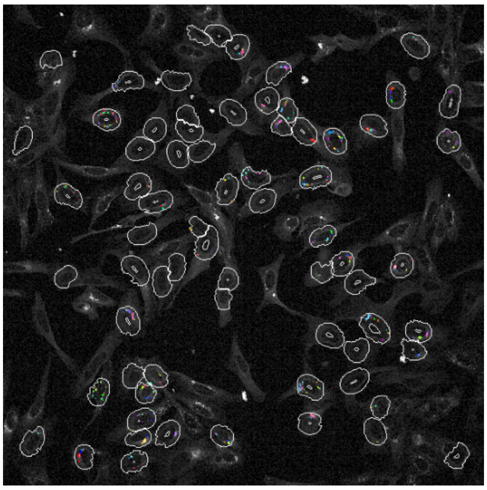<br>White border: Perinuclear ring (from building block 17)<br>Rainbow: Detected RFP spots

24. Find intermediate RFP spots

Input	Method	Output	Example segmented images
Channel:<br>PTS-RFP-FKBP<br><br>Nuclei:<br>GFP and RFP positive cells	Method: D<br><br>Detection sensitivity: 0.75<br><br>Spitting sensitivity: 0.04<br><br>Background correction: 0.5	Population:<br>Intermediate RFP spots	NTC:

(from building block 10)<br><br>Region: Intermediate ring			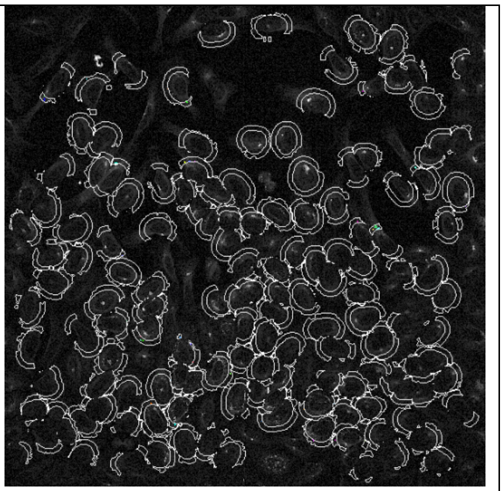<br><br>*crLIS1*:<br>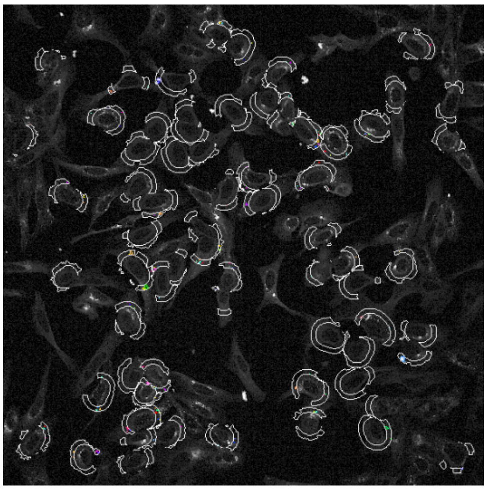<br>White border: Intermediate ring (from building block 18)<br>Rainbow: Detected RFP spots

25. Find outer RFP spots

Input	Method	Output	Example segmented images
Channel: PTS-RFP-FKBP<br><br>Nuclei: GFP and RFP positive cells (from building block 10)<br><br>Region: Outer ring	Method: D<br><br>Detection sensitivity: 0.75<br><br>Spitting sensitivity: 0.04<br><br>Background correction: 0.5	Population: Outer RFP spots	NTC:<br>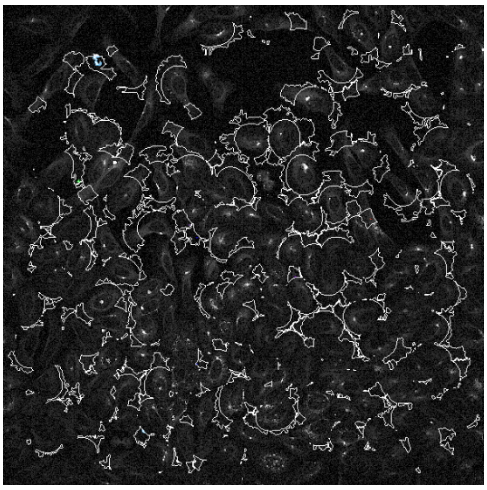

			crLIS1:<br>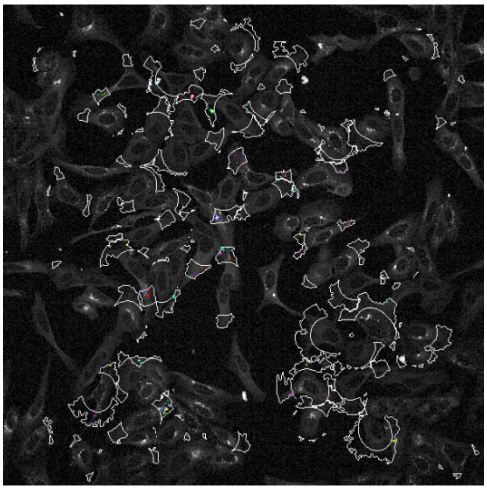<br>White border: Outer ring (from building block 19)<br>Rainbow: Detected RFP spots<br><br>[Summary of all 3 rings]<br>NTC:<br>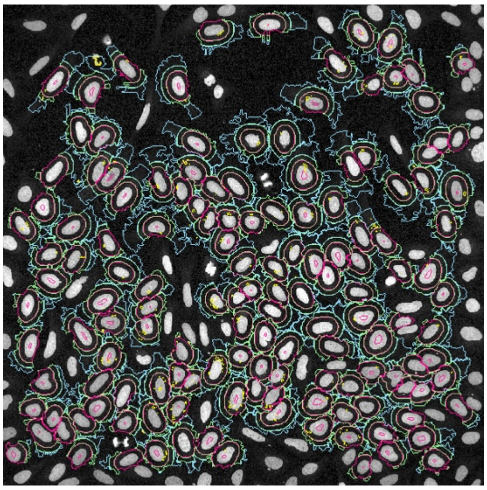<br><br>*crLIS1*:<br>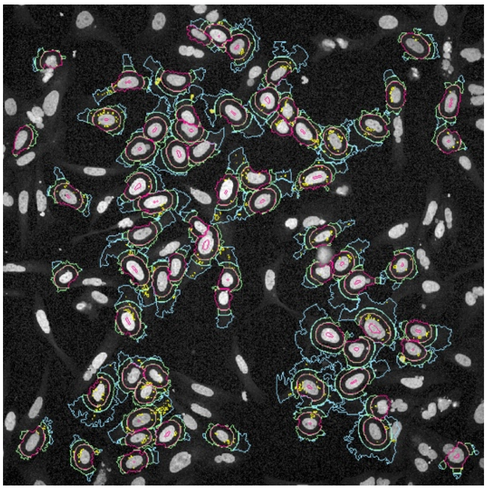<br>Coloured border: Perinuclear (Magenta), Intermediate (Green), Outer (Blue) rings

			Yellow: Detected GFP spots

26. Calculate nucleus texture properties

Input	Method	Output	Example segmented images
Channel: Hoechst<br><br>Nuclei: Viable cells (from building block 6)<br><br>Region: Nucleus	Method: SER features<br><br>Scale: 0.65 μm<br><br>Normalisation by: region intensity	Feature(s):<br>• SER Spot<br>• SER Hole<br>• SER Edge<br>• SER Valley<br>• SER Saddle<br>• SER Bright<br>• SER Dark	N/A

27. Calculate α-tubulin intensity

Input	Method	Output	Example segmented images
Channel: α-tubulin<br><br>Nuclei: Viable cells (from building block 6)<br><br>Region: Cell	Method: Standard	Feature(s): α-tubulin mean intensity	N/A

28. Calculate α-tubulin texture properties

Input	Method	Output	Example segmented images
Channel: α-tubulin<br><br>Nuclei: Viable cells (from building block 6)<br><br>Region: Cell	Method: SER features<br><br>Scale: 0 px<br><br>Normalisation by: Kernel (default normalisation)	Feature(s):<br>• SER Spot<br>• SER Hole<br>• SER Edge<br>• SER Valley<br>• SER Saddle<br>• SER Bright<br>• SER Dark	N/A

29. Calculate EEA1 intensity

Input	Method	Output	Example segmented images
Channel: EEA1<br><br>Nuclei: Viable cells (from building block 6)<br><br>Region: Cell	Method: Standard	Feature(s): EEA1 mean intensity	N/A

30. Calculate EEA1 texture properties

Input	Method	Output	Example segmented images
Channel: EEA1<br><br>Nuclei: Viable cells (from building block 6)<br><br>Region: Cell	Method: SER features<br><br>Scale: 0.65 μm<br><br>Normalisation by: Region intensity	Feature(s):<br>• SER spot<br>• SER Hole<br>• SER Edge<br>• SER Valley<br>• SER Saddle<br>• SER Bright<br>• SER Dark	N/A

31. Calculate EEA1 morphological properties

Input	Method	Output	Example segmented images
Channel: EEA1<br><br>Nuclei: Viable cells (from building block 6)<br><br>Region: Cell	Method: STAR<br><br>Symmetry<br>Radial mean ratio<br>Profile inner nuclear membrane<br>Profile width : 4 pixels<br><br>Sliding parabola curvature: 40	Feature(s):<br>• Symmetry<br>• Radial mean ratio<br>• Profile inner nuclear membrane	N/A

Symmetry properties are calculated via a selected set of polynomial functions.

Radial mean ratio is the mean radius on a filtered image divided by a reference radius (mean radius calculated on the original image).

Profile properties - Intensity of a profile image is a function of the closest distance from a given pixel to a cell border. Profile image is used to characterise the intensity of an input image but only in the location where the profile image is bright – this serves as a weight function when the mean intensity of an input image is calculated. Profile properties are unitless (achieved by dividing the profile-weighted mean intensity by the reference intensity). Profiling the inner nuclear membrane was selected as it gave the most robust separation between NTC versus *crLIS1* based on rZ score.

32. Select cell region (Perinuclear ring for EEA1 spots)

Input	Method	Output	Example segmented images
Nuclei: Viable cells (from building block 6)	Method: Resize region [%]<br><br>Region type: Ring region<br><br>Outer border: 20%<br>Inner border: 100%	Region: Perinuclear ring for EEA1	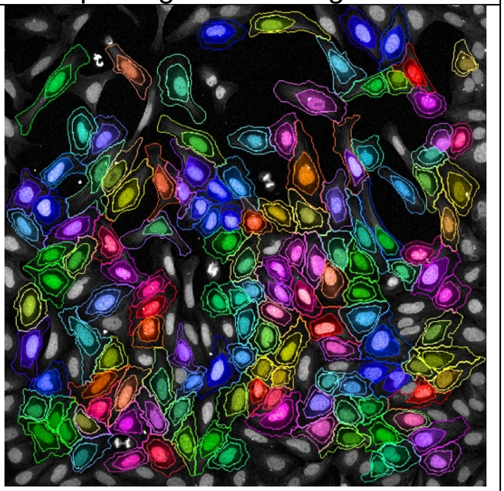<br><br>Rainbow-highlighted: Perinuclear ring (cell boundary also shown)

Border position of 100% corresponds to centre of nucleus. Border position of 0% corresponds to cell boundary.

33. Select cell region (Peripheral ring for EEA1 spots)

Input	Method	Output	Example segmented images
Nuclei: Viable cells (from building block 6)	Method: Resize region [%]<br><br>Region type: Ring region<br><br>Outer border: -20%<br>Inner border: 20%	Region: Peripheral ring for EEA1	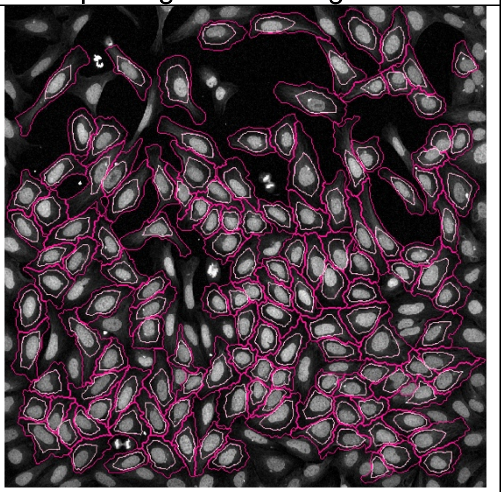<br>Magenta: Outer border of peripheral ring

			White: Inner border of peripheral ring

Outer border was extended to -20% to account for any puncta in protrusions.

34. Find total EEA1 spots

Input	Method	Output	Example segmented images
Channel:<br>EEA1<br><br>Nuclei:<br>Viable cells (from building block 6)<br><br>Region:<br>Cell	Method: B<br><br>Detection sensitivity: 0.85<br><br>Spitting sensitivity: 0.945<br><br>Calculate spot properties	Population:<br>EEA1 spots<br><br>Feature(s):<br>• Total number of EEA1 spots<br>• EEA1 total area<br>• EEA1 region intensity<br>• EEA1 spot to region intensity<br>• EEA1 relative spot intensity	EEA1 channel:<br>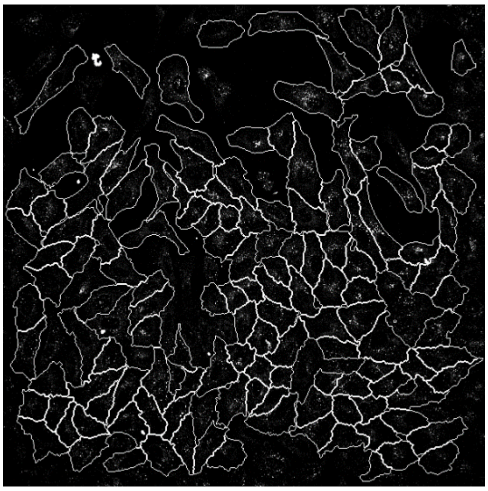<br><br>Detected EEA1:<br>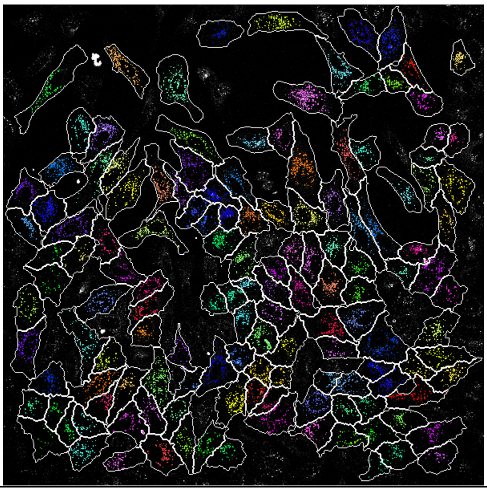

35. Find perinuclear EEA1 spots

Input	Method	Output	Example segmented images
Channel:<br>EEA1<br><br>Nuclei:<br>Viable cells (from building block 6)<br><br>Region:	Method: B<br><br>Detection sensitivity: 0.85<br><br>Spitting sensitivity: 0.945<br><br>Calculate spot properties	Population:<br>Perinuclear EEA1 spots<br><br>Feature(s):<br>• Number of EEA1 spots perinuclear ring	Detected EEA1 spots:<br><br>NTC:

Perinuclear ring for EEA1		• EEA1 perinuclear relative spot intensity	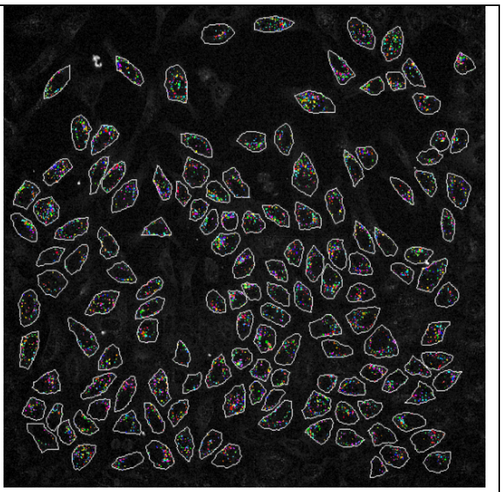<br>*crLIS1 (Decreased number of spots compared to NTC due to dispersal of early endosomes)*<br>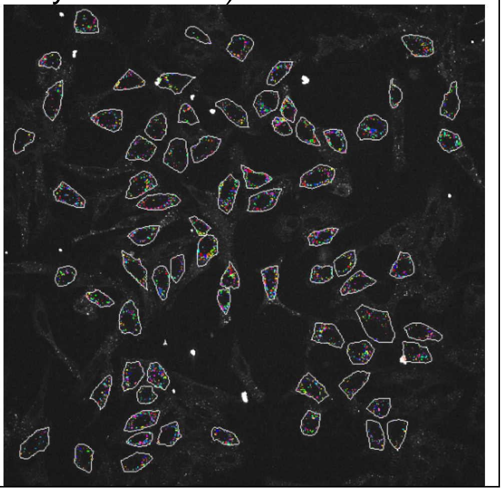

36. Find peripheral EEA1 spots

Input	Method	Output	Example segmented images
Channel: EEA1<br><br>Nuclei: Viable cells (from building block 6)<br><br>Region: Peripheral ring for EEA1	Method: B<br><br>Detection sensitivity: 0.85<br><br>Spitting sensitivity: 0.945<br><br>Calculate spot properties	Population: Peripheral EEA1 spots<br><br>Feature(s):<br>• Number of EEA1 spots peripheral ring<br>• EEA1 peripheral relative spot intensity	Detected EEA1 spots:<br><br>NTC:<br>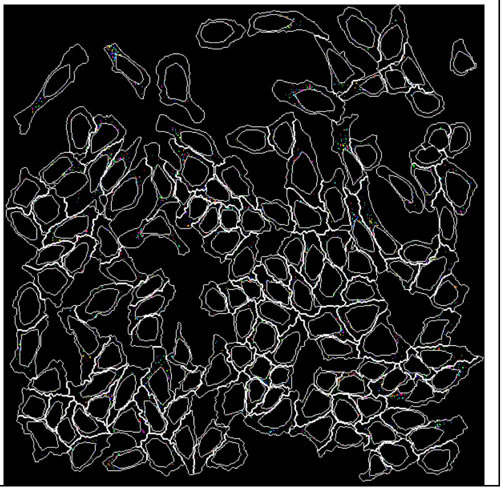

			crLIS1 (Increased number of spots compared to NTC due to dispersal of early endosomes): 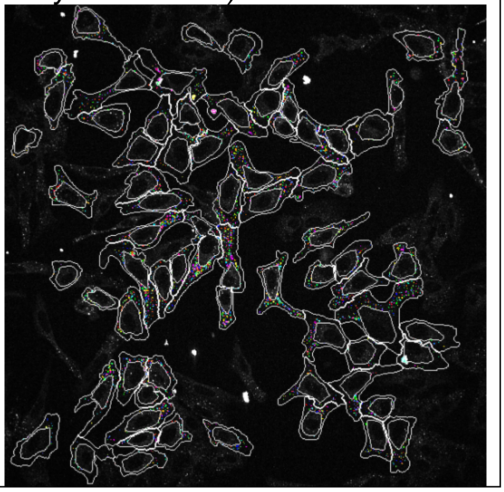

37. Calculate EEA1 localisation ratio

Aim: Calculate relative ratio of EEA1 spots based on their location

Input	Method	Output	Example segmented images
Nuclei: Viable cells (from building block 6)	Method: By Formula<br><br>Formula: A/B<br><br>• Variable A: Number of EEA1 spots peripheral ring<br>• Variable B: Number of EEA1 spots peripheral ring	Feature(s): EEA1 localisation ratio	N/A

38. Calculate EEA1 spot morphology

Input	Method	Output	Example segmented images
Channel: EEA1<br><br>Nuclei: Viable cells (from building block 6)	Method: Standard	Feature(s):<br>• Area<br>• Roundness<br>• Width<br>• Length<br>• Ratio Width to Length	N/A

Region: Total EEA1 spots			

39. Select cells for micronuclei analysis

Aim: A lenient gating for cells excluding debris and mitotic cells

Input	Method	Output	Example segmented images
Nuclei: Nuclei initial removed border (from building block 3)	Method: Filter by property<br><br>Nucleus area (μm^2): ≥ 200	Population: Nuclei for micronuclei	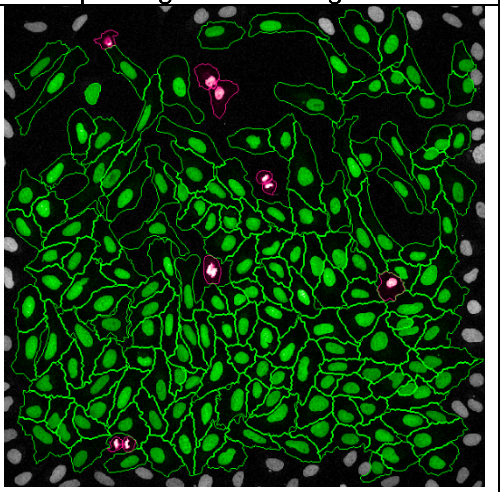<br>Green: selected nuclei<br>Magenta: excluded nuclei

40. Find micronuclei

Aim: Identifying micronucleus – small region on the image having a higher intensity than its surrounding and located outside the nucleus.

Input	Method	Output	Example segmented images
Nuclei: Nuclei for micronuclei (from building block 39)<br><br>Region: Cell	Method: A<br><br>Micronucleus to cytoplasm intensity : > 0.165 (Determines how intense a spot must be to be detected)<br><br>Calculate micronuclei properites: Checked for detection and calculation	Population: Micronuclei<br><br>Feature(s):<br>• Number of micronuclei per cell<br>• Micronuclei fraction of nucleus intensity<br>• Micronuclei relative intensity to border<br>• Micronuclei distance from nucleus	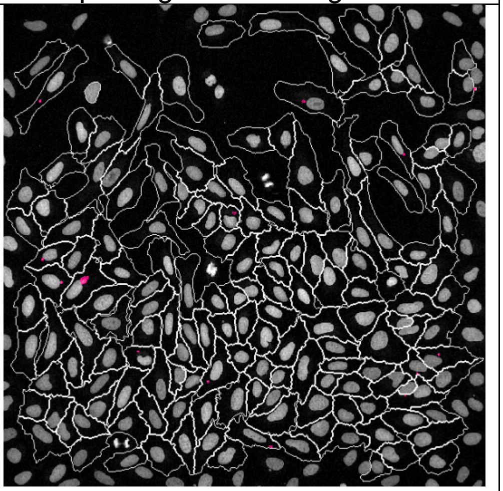<br>Magenta: Segmented micronuclei

		• Micronuclei gap to nucleus<br>• Area of micronucleus<br>• Micronuclei fraction of nucleus area<br>• Micronuclei compactness<br>• Micronuclei to cytoplasm intensity	

- *Micronuclei fraction of nucleus intensity*: Ratio of the micronucleus to nucleus mean intensity
- *Micronuclei relative intensity to border*: mean intensity above the mean border intensity divided by mean border intensity
- *Miconuclei distance from nucleus*: distance from the detected micronucleus centre to the nucleus border
- *Miconuclei gap to nucleus:* minimal distance between border pixels of the micronucleus and the nucleus
- *Micronuclei fraction of nucleus area*: ratio of the micronucleus area to the nucleus area
- *Micronuclei compactness:* Area of the maximum size disk fitting into the micronucleus divided by the area of the entire micronucleus

41. Identify micronuclei positive cells

Aim: Identify population of cells with micronuclei

Input	Method	Output	Example segmented images
Nuclei: Nuclei for micronuclei (from building block 39)	Method: Filter by property<br><br>Number of micronuclei : ≥ 1	Population: Micronuclei positive cells<br><br>Feature(s): Number of micronuclei positive cells	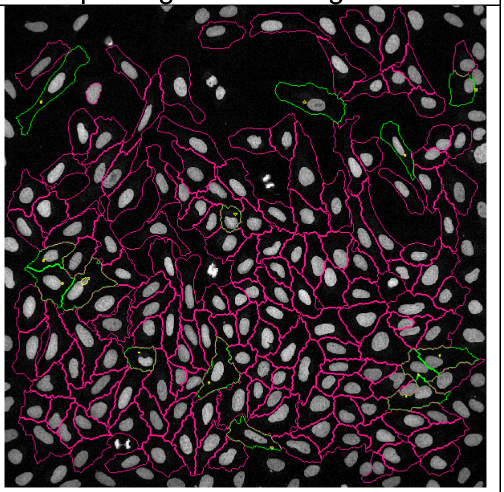<br>Green: Micronuclei positive cells<br>Magenta: Micronuclei negative cells

			crLIS1 (increased number of micronuclei positive cells compared to NTC): 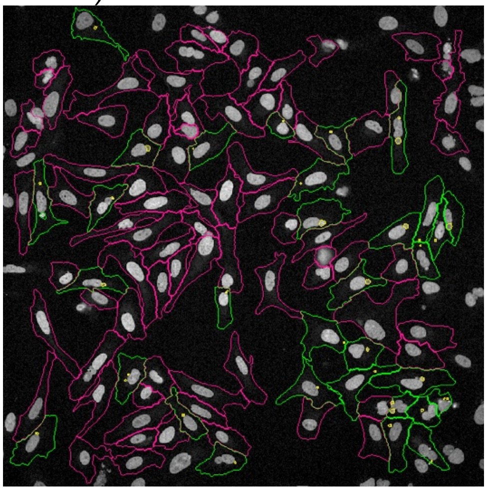 Green: Micronuclei positive cells<br>Magenta: Micronuclei negative cells<br>Yellow: Segmented micronuclei
